# Supplementary material for: Genetic polymorphism in C3 is associated with progression in chronic kidney disease (CKD) patients with IgA nephropathy but not in other causes of CKD
Source: PLoS One. 2020 Jan 31;15(1):e0228101. doi: 10.1371/journal.pone.0228101 (PMC6994105; doi:10.1371/journal.pone.0228101)
Supplement: S1 Table — (DOCX) [file pone.0228101.s001.docx]

**S1 Table. Calculation to determine whether observed genotype frequencies are consistent with Hardy-Weinberg equation**

| ***Complement 3 (rs2230199)*** | **Observed** | **Expected** | **X^2^** | **p-Value** |
| --- | --- | --- | --- | --- |
| **CKD (n= 514)** |  |  |  |  |
| ***FF*** | 48 | 34 |  |  |
| ***FS*** | 168 | 196 | 4.88 | 0.09 |
| ***SS*** | 298 | 284 |  |  |
| **Healthy controls (n=454)** |  |  |  |  |
| ***FF*** | 26 | 19 |  |  |
| ***FS*** | 135 | 149 | 1.86 | 0.39 |
| ***SS*** | 293 | 286 |  |  |

P-Value (Chi-square test), X^2^- Chi-square.

FF-homozygous complement 3 fast, FS-heterozygous complement 3, SS-homozygous complement 3 slow
