# Supplementary material for: Genetic polymorphism in C3 is associated with progression in chronic kidney disease (CKD) patients with IgA nephropathy but not in other causes of CKD
Source: PLoS One. 2020 Jan 31;15(1):e0228101. doi: 10.1371/journal.pone.0228101 (PMC6994105; doi:10.1371/journal.pone.0228101)
Supplement: S2 Table — (DOCX) [file pone.0228101.s002.docx]

**S2 Table. Comparison of baseline characteristics (clinical and biochemical) between CKD patients with the *C3FF*, and those with *C3FS or SS*.**

|  | ***Complement 3 FF* (n=48)** | ***Complement 3 FS/SS***  **(n=466)** | **p-Value** |
| --- | --- | --- | --- |
| **Age, years** | 63.3 (50.6 – 74.8) | 62.8 (50.4 – 73.5) | 0.64 |
| **Gender (male), n (%)** | 31 (64.6%) | 291 (62.4%) | 0.77 |
| **Ethnicity (Caucasian), n (%)** | 48 (100%) | 446 (95.7%) | 0.14 |
| **Smoking, n (%)** | 30 (62.5%) | 293 (62.9%) | 0.96 |
| **HTN, n (%)** | 46 (95.8%) | 449 (96.4%) | 0.86 |
| **DM, n (%)** | 16 (33.3%) | 142 (30.5%) | 0.74 |
| **Tumor, n (%)** | 4 (8.3%) | 48 (10.3%) | 0.67 |
| **eGFR (CKD-EPI)** | 31.8 (22.6 – 43.2) | 27.5 (19.8 – 36.6) | **0.029** |
| **Delta eGFR (ml/min/1.73m^2^/year)** | -3.2 (-4.9 to 0.02) | -0.47 (-4.6 to 0.08) | 0.53 |
| **Albumin (g/L)** | 42 (40.5 – 44) | 43 (40 – 45) | 0.17 |
| **Corrected calcium (mmol/L)** | 2.3 (2.2 – 2.4) | 2.2 (2.2 – 2.4) | 0.82 |
| **Phosphorus (mmol/L)** | 1.1 (1.0 – 1.2) | 1.1 (0.98 – 1.3) | 0.87 |
| **PTH (pmol/L)** | 6.8 (3.9 – 12.3) | 6.9 (4.1 – 11.0) | 0.81 |
| **uPCR (g/mol)** | 51.6 (14 – 189) | 33.8 (14 – 133) | 0.58 |
| **Haemoglobin (g/L)** | 125 (116.5 – 136) | 124 (114 – 136) | 0.84 |
| **CRP (mg/L)** | 4.2 (2.0 – 8.1) | 2.6 (1.1 – 5.3) | **0.006** |
| **Cholesterol(mmol/L)** | 4.7 (3.8 – 5.5) | 4.5 (3.7 – 5.4) | 0.51 |

HTN-hypertension, DM-diabetes mellitus, eGFR-estimated glomerular filtration rate calculated using CKD-EPI equation, PTH-parathyroid hormone, uPCR-urine protein:creatinine ratio, CRP- C-reactive protein.

Continuous variables are expressed as median (interquartile range), p-Value by Man-Whitney U test.

Categorical variables are expressed as number (%), p-Value by Chi-Square test.
