# Supplementary material for: Genetic polymorphism in C3 is associated with progression in chronic kidney disease (CKD) patients with IgA nephropathy but not in other causes of CKD
Source: PLoS One. 2020 Jan 31;15(1):e0228101. doi: 10.1371/journal.pone.0228101 (PMC6994105; doi:10.1371/journal.pone.0228101)
Supplement: S3 Table — (DOCX) [file pone.0228101.s003.docx]

**S3 Table. Comparison of allele frequency between rapid and stable CKD patients in different causes of CKD**

| **Cause of CKD** | **Stable patients**  **(n=255)** | **Rapid progressors**  **(n=259)** | **p-Value*** |
| --- | --- | --- | --- |
| ***Complement 3***  ***(rs2230199) Allele frequency*** |  |  |  |
| **DM (n=101)** | 51 | 50 |  |
| ***F*** | 26 (25.5%) | 32 (32%) | 0.35 |
| ***S*** | 76 (74.5%) | 68 (68%) |  |
| **HTN (n=55)** | 34 | 21 |  |
| ***F*** | 17 (25%) | 7 (16.7%) | 0.35 |
| ***S*** | 51 (75%) | 35 (83.3%) |  |
| **RVD (n=32)** | 20 | 12 |  |
| ***F*** | 16 (40%) | 7 (29.2%) | 0.43 |
| ***S*** | 24 (60%) | 17 (70.8%) |  |
| **IgA nephropathy (n=37)** | 16 | 21 |  |
| ***F*** | 3 (9.4%) | 17 (40.5%) | **0.003** |
| ***S*** | 29 (90.6%) | 25 (59.5%) |  |
| **FSGS (n=16)** | 5 | 11 |  |
| ***F*** | 1 (10%) | 8 (36.4%) | 0.21 |
| ***S*** | 9 (90%) | 14 (63.6%) |  |
| **Membranous (n=12)** | 5 | 7 |  |
| ***F*** | 2 (20%) | 1 (7.1%) | 0.55 |
| ***S*** | 8 (80%) | 13 (92.9%) |  |
| **Other GN & vasculitis (n=33)** | 17 | 16 |  |
| ***F*** | 8 (23.5%) | 7 (21.9%) | 1.00 |
| ***S*** | 26 (76.5%) | 25 (78.1%) |  |
| **APKD (n=57)** | 2 | 55 |  |
| ***F*** | 3 (75%) | 36 (32.7%) | 0.12 |
| ***S*** | 1 (25%) | 74 (67.3%) |  |
| **Pyelo & interstitial nephritis (n=67)** | 42 | 25 |  |
| ***F*** | 17 (20.2%) | 7 (14%) | 0.49 |
| ***S*** | 67 (79.8%) | 43 (86%) |  |
| **Unknown (n=60)** | 38 | 22 |  |
| ***F*** | 18 (23.7%) | 12 (27.3%) | 0.67 |
| ***S*** | 58 (76.3%) | 32 (72.7%) |  |
| **Others (n=44)** | 25 | 19 |  |
| ***F*** | 13 (26%) | 6 (15.8%) | 0.30 |
| ***S*** | 37 (74%) | 32 (84.2%) |  |

DM-diabetes mellitus, HTN-hypertension, RVD-renovascular disease, FSGS- focal segmental glomerular sclerosis, GN-glomerulonephritis, ADPKD-autosomal dominant polycystic kidney disease, F complement 3 fast, and S complement 3 slow.

*p-Value for allele frequency by Fisher-exact test
