# Supplementary material for: Genetic polymorphism in C3 is associated with progression in chronic kidney disease (CKD) patients with IgA nephropathy but not in other causes of CKD
Source: PLoS One. 2020 Jan 31;15(1):e0228101. doi: 10.1371/journal.pone.0228101 (PMC6994105; doi:10.1371/journal.pone.0228101)
Supplement: S4 Table — (DOCX) [file pone.0228101.s004.docx]

**S4 Table. Comparison of the clinical and biochemical characteristics between IgAN patients with either RP or SP**

|  | **Stable patients**  **(n=16)** | **Rapid progressors**  **(n=21)** | **P-value** |
| --- | --- | --- | --- |
| **Age (years)** | 53.5 (47.1 – 61.2) | 52.3 (38.2 – 64.8) | 0.77 |
| **Gender (male), n (%)** | 13 (81.3%) | 14 (66.7%) | 0.46 |
| **Ethnicity (Caucasian)** | 15 (93.8%) | 20 (95.2%) | 1.00 |
| **Smoking (smokers)** | 8 (50%) | 11 (52.4%) | 1.00 |
| **HTN, n (%)** | 16 (100%) | 21 (100%) |  |
| **SBP (mmHg)** | 137.5 (123 – 146.5) | 133 (120 – 150) | 0.82 |
| **DBP (mmHg)** | 76 (72 – 80) | 76 (72 – 80) | 0.82 |
| **DM, n (%)** | 1 (6.3%) | 2 (9.5%) | 1.00 |
| **Tumor, n (%)** | 1 (6.3%) | 1 (4.8%) | 1.00 |
| **MI, n (%)** | 0 (0.0%) | 1(4.8%) | 1.00 |
| **PVD, n (%)** | 16 (100%) | 21 (100%) |  |
| **CVA, n (%)** | 0 (0.0%) | 1 (4.8%) | 1.00 |
| **CCF, n (%)** | 0 (0.0%) | 1 (4.8%) | 1.00 |
| **Creatinine (umol/L)** | 188.5 (147.5 – 245) | 161 (128 – 197) | 0.32 |
| **eGFR (CKD-EPI)** | 29.6 (21.3 – 41.7) | 37 (31.4 – 49) | 0.17 |
| **Delta eGFR (ml/min/year)** | 0.13 (-0.2 to 0.34) | -6 (-4.5 to -6.7) | **˂0.001** |
| **Urea (mmol/L)** | 13.5 (10.7 – 17.6) | 11.8 (8.9 – 15.6) | 0.32 |
| **Albumin (g/L)** | 43.5 (42 – 44.5) | 40 (39 – 41) | **0.004** |
| **Corrected Calcium (mmol/L)** | 2.3 (2.25 – 2.4) | 2.3 (2.2 – 2.38) | 0.75 |
| **Phosphorus (mmol/L)** | 1.1 (0.99 – 1.2) | 1.2 (1.1 – 1.3) | 0.27 |
| **PTH (pmol/L)** | 4.8 (3.2 – 7.6) | 5 (3.4 – 8) | 0.68 |
| **Vit D^a^ (nmol/L)** | 29.4 (15.3 – 41.8) | 48.7 (33.2 – 75.4) | **0.04** |
| **CRP (mg/L)** | 1.8 (1 – 2.9) | 3.3 (2.6 – 5.8) | 0.07 |
| **UPCR (g/mol)** | 28.4 (17.5 – 48.6) | 182 (60 – 223) | **˂0.001** |
| **Urate^b^ (mmol/L)** | 0.51 (0.46 – 0.54) | 0.45 (0.37 – 0.57) | 0.29 |
| **T.Cholesterol(mmol/L)** | 4.3 (3.9 – 4.6) | 4.8 (4.4 – 5.2) | **0.04** |
| **Haemoglobin (g/L)** | 132.5 (118.5 – 137) | 134 (124 – 141) | 0.58 |
| **HbA1c^c^  (mmol/mol)** | 37.5 (35 – 43.5) | 39 (37 – 41.4) | 0.66 |

HTN-hypertension, BP-blood pressure, DM-diabetes mellitus, MI-myocardial infarction, CCF-congestive cardiac failure, PVD-peripheral vascular disease, CVA-cerebrovascular accident, eGFR-estimated glomerular filtration rate calculated using CKD-EPI equation, PTH-parathyroid hormone, CRP- C-reactive protein, uPCR-urine protein:creatinine ratio, HbA1c-haemoglobin A1c.

Continuous variables are expressed as median (interquartile range) and p-Value by Man-Whitney U test.

Categorical variables are expressed as number (%) and p-Value by exact fisher test.

a- Vit D results were only available in 9 (56.3%) of slow progression and 10 (47.6%) of rapid progression

b-Urate results were only available in 12 (75%) of slow progression and 18 (85.7%) of rapid progression

c-HbA1C were only available in 12 (75%) of slow progression and 18 (85.7%) of rapid progression
