# Supplementary material for: Genetic polymorphism in C3 is associated with progression in chronic kidney disease (CKD) patients with IgA nephropathy but not in other causes of CKD
Source: PLoS One. 2020 Jan 31;15(1):e0228101. doi: 10.1371/journal.pone.0228101 (PMC6994105; doi:10.1371/journal.pone.0228101)
Supplement: S5 Table — (DOCX) [file pone.0228101.s005.docx]

**S5 Table. Cox regression analysis (renal progression) univariate and multivariate models (CKD cohort n = 514, events n = 261)**

| **Factor** | **Univariate model**  **HR (95% CI)** | **p-Value** | **Multivariate Model**  **HR (95% CI)** | **p-Value** |
| --- | --- | --- | --- | --- |
| ***Complement 3 FF*** | 1.1 (0.75 – 1.6) | 0.59 | 1.2 (0.78 – 1.8) | 0.40 |
| ***Complement 3 FS*** | 1.0 (0.8 – 1.3) | 0.78 | 1.1 (0.82 – 1.4) | 0.59 |
| ***Complement 3 SS*** | 0.95 (0.74 – 1.2) | 0.70 |  |  |
| **Age** | 0.98 (0.97 – 0.99) | **<0.001** | 0.98 (0.97 – 0.99) | **<0.001** |
| **Gender (female)** | 1.4 (1.1 – 1.8) | **0.003** | 1.5 (1.1 – 2.0) | **0.005** |
| **Smoking** | 1.1 (0.8 – 1.4) | 0.45 | 1.5 (1.1 – 1.9) | **0.004** |
| **HTN** | 1.1 (0.58 – 2.2) | 0.70 | 1.3 (0.64 – 2.5) | 0.49 |
| **DM** | 0.89 (0.67 – 1.17) | 0.41 | 0.9 (0.67 – 1.2) | 0.52 |
| **eGFR (CKD-EPI)** | 0.99 (0.98 – 1.00) | 0.17 | 0.98 (97 – 99) | **0.012** |
| **Albumin (g/L)** | 0.94 (0.92 – 0.96) | **<0.001** | 0.98 (0.94 – 1.0) | 0.26 |
| **Haemoglobin (g/L)** | 0.98 (0.97 – 0.99) | **<0.001** | 0.98 (0.97 – 0.99) | **0.002** |
| **UPCR (g/mol)** | 1.01 (1.01 – 1.02) | **<0.001** | 1.01 (1.0 – 1.02) | **0.002** |

FF-homozygous complement 3 fast, FS-heterozygous complement 3, HTN-hypertension, DM-diabetes mellitus, eGFR-estimated glomerular filtration rate calculated using CKD-EPI equation, uPCR-urine protein:creatinine ratio.
