# Supplementary material for: Genetic polymorphism in C3 is associated with progression in chronic kidney disease (CKD) patients with IgA nephropathy but not in other causes of CKD
Source: PLoS One. 2020 Jan 31;15(1):e0228101. doi: 10.1371/journal.pone.0228101 (PMC6994105; doi:10.1371/journal.pone.0228101)
Supplement: S6 Table — (DOCX) [file pone.0228101.s006.docx]

**S6 Table. Baseline characteristics (clinical and biochemical) of the different GN groups (total n=269)**

|  | **Total GN**  **(n=269)** | **IgA nephropathy**  **(n=114)** | **FSGS**  **(n=50)** | **Membranous nephropathy**  **(n=59)** | **Other GN**  **(n=46)** |
| --- | --- | --- | --- | --- | --- |
| **Age, years** | 59.9  (47.9 -68.5) | 56.5  (45 – 68) | 55  (43 – 64) | 68  (58 – 74) | 64  (49 – 68.5) |
| **Gender (male), n (%)** | 195(72.5%) | 94 (82.5%) | 30 (60%) | 42 (71.2%) | 29 963%) |
| **Ethnicity (Caucasian), n (%)** | 267(99.3%) | 112 (98.3%) | 50 (100%) | 59 (100%) | 46 (100%) |
| **Smoking, n (%)** | 163(60.6%) | 66 (58%) | 34 (68%) | 34 (57.6%) | 29 (63%) |
| **HTN, n (%)** | 248(92.2%) | 108 (94.7%) | 47 (94%) | 53 (89.8%) | 40 (87%) |
| **DM, n (%)** | 42(15.6%) | 12 (10.5%) | 7 (14 %) | 15 (25.4%) | 8 (17.4%) |
| **Tumour, n (%)** | 27(10%) | 6 (5.3%) | 9 (18%) | 7 (11.9%) | 5 (10.9%) |
| **Conservative treatment, n (%)** | 168(62.5%) | 88 (77.2%) | 31 (62%) | 34 (57.6%) | 15 (32.6%) |
| **Corticosteroids ± Immunosupression** | 101(37.5%) | 26 (22.8%) | 19 (38%) | 25 (42.4%) | 31 (67.4%) |
| **eGFR(CKD-EPI)**  **(ml/min/1.73m^2^)** | 33.5  (21.3-46.6) | 28.6  (17 – 42) | 35.9  (26-48.6) | 35  (23.4 – 50) | 35.2  (25.8-49.5) |
| **Delta eGFR (ml/min/1.73m^2^/year)** | -1.6  (-4.2 to 0.06) | -1.8  (-0.3 to -5.0) | -1.9  (0.0 to -4.3) | -1.1  (0.5 to -3.2) | -1.0  (0.9 to -4.2) |
| **Albumin (g/L)** | 41  (38-44) | 42  (40 – 45) | 41  (38 – 44) | 38  (33 – 42) | 41  (37 – 43) |
| **Corrected calcium (mmol/L)** | 2.3  (2.2 - 2.4) | 2.3  (2.2 – 2.4) | 2.3  (2.2 –2.4) | 2.3  (2.2 – 2.4) | 2.3  (2.2 – 2.5) |
| **Phosphorus (mmol/L)** | 1.15  (1.0-1.3) | 1.1  (1.0 – 1.3) | 1.2  (1.0 – 1.3) | 1.2  (1.0 – 1.3) | 1.2  (0.9 – 1.3) |
| **PTH (pmol/L)** | 5.5  (3.1-8.9) | 6.1  (3.2 – 10.6) | 4.8  (5.6 – 7.3) | 5.5  (3.0 – 8.8) | 5.3  (3.0 – 7.2) |
| **uPCR (g/mol)** | 91  (27-241) | 75  (32 – 185) | 91  (23 – 293) | 170  (52 – 479) | 62  (16 – 211) |
| **Haemoglobin (g/L)** | 125  (114-137) | 127  (116-137) | 128  (115-143) | 117  (108-128) | 126  (115-138) |

HTN-hypertension, DM-diabetes mellitus, FSGS- focal segmental glomerular sclerosis, GN-glomerulonephritis, eGFR-estimated glomerular filtration rate calculated using CKD-EPI equation, PTH-parathyroid hormone, uPCR-urine protein:creatinine ratio. Continuous variables are expressed as median (interquartile range).

Categorical variables are expressed as number (%).
