# Supplementary material for: Genetic polymorphism in C3 is associated with progression in chronic kidney disease (CKD) patients with IgA nephropathy but not in other causes of CKD
Source: PLoS One. 2020 Jan 31;15(1):e0228101. doi: 10.1371/journal.pone.0228101 (PMC6994105; doi:10.1371/journal.pone.0228101)
Supplement: Attachment — Submitted filename: Response to reviewers.docx [file pone.0228101.s007.docx]

**Response to reviewers**

Dear editor and reviewers,

Thank you so much for reviewing our manuscript and providing us with your comments and queries, aiming to improve our work. The following is our reply to your comments and queries.

1- **Concern about the novelty regarding the association of the R102G and CKD**

The association between the R102G polymorphism of C3 and CKD or GN is not a novel one and it has been addressed by some studies before. This is the main point we have relied upon in the rationale of our study that the R102G may be associated with progression of all-cause CKD or in specific sub-types of GN (hence study 2 which included all the biopsy proven GN patients in our cohort). The novel point in our study is the association between the R102G and progression in IgA nephropathy patients. This association has not been addressed by any of the previous studies.

-We have now illustrated this point in a clearer way in the introduction and the discussion of the revised manuscript.

2- **Need to analyze the results of the study considering the already available GWAS data on CKD**

Most available GWAS that have been conducted in CKD [1-5] or IgA nephropathy [6-8] patients aimed to investigate the genetic factors associated with prevalent CKD or IgAN; as these studies lacked the detailed clinical characteristics of the patients and follow up data they were unable to include progression as an end-point. The only 2 GWAS that attempted to search for genetic factors associated with progression rather than prevalence of CKD are Boger CA et al [9] and Parsa A et al [10].Boger et al`s GWAS tested only 16 SNPs and found that 11 of them associated with incident CKD (during a follow up period of 7 years). Our targeted SNP, the R102G, was not one of the 16 tested SNPs in this GWAS. Parsa et al`s GWAS tested a million SNPs in a large CKD cohort with 5 years follow up and found 12 SNPs that were associated with time to ESRD in black patients and 6 in white patients; the R102G SNP was not one of the latter 6 SNPs and this agrees with our finding that the R102G was not associated with progression in all-cause CKD. However in Pars et al`s study the specific causes of CKD in their cohort were not defined and so they could not relate primary renal disease-specific progression to specific SNPs.

-We have added a paragraph about this in our discussion in the revised manuscript.

3- **Concern about the sample size of the 37 patients with IgA nephropathy too small to be convincing of a different distribution of the polymorphism on the RP and SP groups**

This is correct and we mentioned this point in our manuscript`s discussion as a limitation. However in study 2 with larger number of IgAN patients (114) we found that C3F is strongly and independently associated with the progression in IgAN patients. We believe that our study could underpin future studies in larger cohorts of IgAN patients which could then replicate this association.

4- **Need to evaluate the pathophysiological link between the R102G polymorphism and complement activation**

The pathophysiological link between the R102G and complement activation has been evaluated previously by Heurich M et al [11]. They used C3FF and C3SS from plasma of healthy individuals and added them separately to antibody coated sheep RBCs and then added factor B (FB) to them. They found that the samples containing C3SS needed larger amount of FB to lyse the RBCs. Demonstrating that plasma containing C3FF has more complement activity than that containing C3SS. They further investigated this issue and proved that factor H (one of the complement regulator proteins) bound less well to C3FF than C3SS.

-We have added a paragraph about this issue in the introduction of the revised manuscript.

5- **Need to include in the multivariate Cox regression model the evaluation of the IgAN subgroup CRP**

-We have added the CRP to the univariate Cox regression in table 6 in the revised manuscript. It showed no significant association with progression in IgAN patients so we have not added it to the multivariate Cox model. The variables which showed significant association in the univariate model are the only variables that were used in the multivariate model to be consistent with the statistical rule that one variable can be included in the Cox model for every 8-10 patients with the event of interest (we had 59 events).

6- **Concern about the fact that the study featured a cohort that was primarily Caucasian which limits the generalization of the results**

All genetic studies should define the ethnicity of the studied cohort according to the prevalence of studied SNPs. If the studied SNP prevalence is ˂ 5% in a certain ethnicity the presence or the absence of the association between this SNP and the disease will not be accurate due to the rarity of the SNP in this ethnicity. The frequency of the C3F allele differs in different ethnicities: Caucasian (20%), black (5%) and Asian (1%) [12]. Hence the Caucasian cohort appears to be the optimal cohort to be used to investigate the R102G SNP in different diseases.

7- **Concern about the significant differences in age between RP and SP likely contributing to findings.**

The difference between the age of patients in RP and SP groups was only significant in the whole CKD cohort but it was not significant in the sub group of IgAN patients (37 patients: 21 RP and 16 SP). Also the age was not significantly associated with progression in the IgAN patients in the Cox regression (table 6). The Cox regression analysis for death was undertaken in the whole cohort without separation into SP and RP; adjustment for age in the multivariate analysis was also performed (table 4).

-We have added the table comparing the baseline characteristics of the IgAN subgroup to the supplementary tables in the revised manuscript (S4 table).

8- **Need to indicate how common the C3F variant is in the general population**

As mentioned above the frequency of the C3F allele differs in different ethnicities (Caucasian (20%), black (5%) and Asian (1%)). We have previously referred to its frequency in Caucasian cohorts and in our healthy control group in the discussion section.

-We have now illustrated this point in the introduction of the revised manuscript.

9- **Concern about the fact that Table 6 shows a regression analysis of the IgAN group in which the multivariate regression analysis did not include the C3SS genotype as a variant.**

Statistically we cannot include C3SS in the same multivariate model with C3FF and C3FS as they are constant or linearly dependent covariates; C3SS = 1- (C3FF + C3FS).

-We have added a second multivariate model to table 6 in the revised manuscript with C3SS (but without C3FF and C3FS) to show that it is still protective of progression in the multivariate model.

All of the above modifications have been highlighted in yellow in the revised manuscript.

Thank you so much,

Sara T Ibrahim

**References**

1. Kottgen A, Glazer NL, Dehghan A, Hwang SJ, Katz R, Li M, et al. Multiple loci associated with indices of renal function and chronic kidney disease. Nature Genetics. 2009;41:712-7.
2. Kottgen A, Pattaro C, Boger CA, Fuchsberger C, Olden M, Glazer NL, et al. New loci associated with kidney function and chronic kidney disease. Nature genetics. 2010;42(5):376-84.
3. Pattaro C, Kottgen A, Teumer A, Garnaas M, Boger CA, Fuchsberger C, et al. Genome-wide association and functional follow-up reveals new loci for kidney function. PLoS genetics. 2012;8(3):e1002584.
4. Pattaro C, Teumer A, Gorski M, Chu AY, Li M, Mijatovic V, et al. Genetic associations at 53 loci highlight cell types and biological pathways relevant for kidney function. Nat Commun. 2016;7:10023.
5. Chambers J, Zhang W, Lord G, van der Harst P, Lawlor DA, Sehmi JS, et al: Genetic loci influencing kidney function and chronic kidney disease. Nat Genet. 2010;42:373-5.
6. Gharavi AG, Kiryluk K, Choi M, Li Y, Hou P, Xie J, et al: Genome-wide association study identifies susceptibility loci for IgA nephropathy. Nat Genet. 2011;43:321-7.
7. Yu XQ, Li M, Zhang H, Low HQ,  Wei X, Wang JQ, et al: A genome-wide association study in Han Chinese identifies multiple susceptibility loci for IgA nephropathy. Nat Genet. 2011;44:178-82.
8. Kiryluk K, Li Y, Scolari F, Sanna-Cherchi S,  Choi M, Verbitsky M, et al: Discovery of new risk loci for IgA nephropathy implicates genes involved in immunity against intestinal pathogens. Nat Genet. 2014;46:1187-96.
9. Böger CA, Gorski M, Li M, Hoffmann MM, Huang C, Yang Q, et al. Association of eGFR-Related Loci Identified by GWAS with Incident CKD and ESRD. PLoS Genet. 2011;7(9):e1002292.
10. Parsa A, Kanetsky PA, Xiao R, Gupta J, Mitra N, Limou S, et al. Genome-Wide Association of CKD Progression: The Chronic Renal Insufficiency Cohort Study. J Am Soc Nephrol. 2017;28(3):923-34.
11. Heurich M, Martínez-Barricarte R, Francis NJ, Roberts DL, Rodríguez de Córdoba S, Morgan BP, et al. Common polymorphisms in C3, factor B, and factor H collaborate to determine systemic complement activity and disease risk. Proc Natl Acad Sci U S A. 2011;108(21):8761–6.
12. Bazyar N, Azarpira N, Khatami RS, Galehdari H. The investigation of allele and genotype frequencies of human C3 (rs2230199). Mol Biol Rep. 2012;39(9):8919–24.
